# Molecular Landscape and Actionable Alterations in a Genomically Guided Cancer Clinical Trial: National Cancer Institute Molecular Analysis for Therapy Choice (NCI-MATCH)

**DOI:** 10.1200/JCO.19.03010

**Published:** 2020-10-13

**Authors:** Keith T. Flaherty, Robert J. Gray, Alice P. Chen, Shuli Li, Lisa M. McShane, David Patton, Stanley R. Hamilton, P. Mickey Williams, A. John Iafrate, Jeffrey Sklar, Edith P. Mitchell, Lyndsay N. Harris, Naoko Takebe, David J. Sims, Brent Coffey, Tony Fu, Mark Routbort, James A. Zwiebel, Larry V. Rubinstein, Richard F. Little, Carlos L. Arteaga, Robert Comis, Jeffrey S. Abrams, Peter J. O’Dwyer, Barbara A. Conley

**Affiliations:** ^1^ Massachusetts General Hospital, Boston, MA; ^2^ ECOG-ACRIN Cancer Research Group Biostatistics Center, Dana Farber Cancer Institute Boston, MA; ^3^ Division of Cancer Treatment and Diagnosis, National Cancer Institute (NCI), National Institutes of Health (NIH), Bethesda, MD; ^4^ Center for Biomedical Informatics and Information Technology, NCI, NIH, Bethesda, MD; ^5^ University of Texas MD Anderson Cancer Center, Houston, TX; ^6^ Frederick National Laboratory for Cancer Research, Frederick, MD; ^7^ Harvard University, Boston, MA; ^8^ Yale University, New Haven, CT; ^9^ Thomas Jefferson University Hospital, Philadelphia, PA; ^10^ Center for Biomedical Informatics and Information Technology, Frederick National Laboratory for Cancer Research, Frederick, MD; ^11^ University of Texas Southwestern Simmons Cancer Center, Dallas, TX; ^12^ ECOG-ACRIN Cancer Research Group, Philadelphia, PA; † Deceased.

## Abstract

Therapeutically actionable molecular alterations are widely distributed across cancer types. The National Cancer Institute Molecular Analysis for Therapy Choice (NCI-MATCH) trial was designed to evaluate targeted therapy antitumor activity in underexplored cancer types. Tumor biopsy specimens were analyzed centrally with next-generation sequencing (NGS) in a master screening protocol. Patients with a tumor molecular alteration addressed by a targeted treatment lacking established efficacy in that tumor type were assigned to 1 of 30 treatments in parallel, single-arm, phase II subprotocols.

**PATIENTS AND METHODS:**

Tumor biopsy specimens from 5,954 patients with refractory malignancies at 1,117 accrual sites were analyzed centrally with NGS and selected immunohistochemistry in a master screening protocol. The treatment-assignment rate to treatment arms was assessed. Molecular alterations in seven tumors profiled in both NCI-MATCH trial and The Cancer Genome Atlas (TCGA) of primary tumors were compared.

**RESULTS:**

Molecular profiling was successful in 93.0% of specimens. An actionable alteration was found in 37.6%. After applying clinical and molecular exclusion criteria, 17.8% were assigned (26.4% could have been assigned if all subprotocols were available simultaneously). Eleven subprotocols reached their accrual goal as of this report. Actionability rates differed among histologies (eg, > 35% for urothelial cancers and < 6% for pancreatic and small-cell lung cancer). Multiple actionable or resistance-conferring tumor mutations were seen in 11.9% and 71.3% of specimens, respectively. Known resistance mutations to targeted therapies were numerically more frequent in NCI-MATCH than TCGA tumors, but not markedly so.

**CONCLUSION:**

We demonstrated feasibility of screening large numbers of patients at numerous accruing sites in a complex trial to test investigational therapies for moderately frequent molecular targets. Co-occurring resistance mutations were common and endorse investigation of combination targeted-therapy regimens.

## INTRODUCTION

The first targeted therapy successes in oncogene-driven cancers were specific to single cancer histologies (eg, *BCR-ABL* translocations in chronic myelogenous leukemia^1^; *ERBB2* gene amplification in breast cancer^2^; *BRAF* mutations in melanoma^3^; and *EGFR* mutations and *ALK* translocations in lung adenocarcinoma^4,5^). BRAF-inhibitor therapy was explored across a spectrum of *BRAF*-mutated cancers and yielded high response rates in melanoma, non–small-cell lung cancer (NSCLC), and Langerhans cell histiocytosis but unanticipated resistance in colorectal cancer, despite ample preclinical evidence favoring efficacy.^6,7^ More recently, the US Food and Drug Administration (FDA) has approved the programmed death-1 inhibitor pembrolizumab for any patient with mismatch repair deficiency and high microsatellite instability. This abnormality occurs in approximately 2% of patients.^8,9^ Larotrectinib was approved for any patient whose tumor harbors a neurotrophic tropomyosin receptor kinase (*NTRK*) fusion, which, although common in several rare tumors, occurs in < 1% of most tumor histologies.^10,11^

**CONTEXT**

**Key Objective**
To determine the likelihood of identifying molecular alterations by next-generation sequencing that points to approved or investigational targeted therapies.
**Knowledge Generated**
In this multiarm phase II trial, all available investigational therapies known with evidence of efficacy in biomarker-defined populations were simultaneously deployed. With centralized molecularly screening performed on freshly procured tumor biopsy specimens, 38% of patients had actionable alterations and 18% were assigned to actively enrolling treatment arms.
**Relevance**
Performance of next-generation sequencing in biopsy specimens from patients with relapsed-refractory advanced permits triaging nearly one-fifth of patients to evidence-based investigational therapy.

The National Cancer Institute–Molecular Analysis for Therapy Choice (NCI-MATCH) trial was the first national-scale trial in the United States incorporating centralized diagnostic testing and geographically distributed clinical investigation of dozens of treatment options in parallel. We hypothesized that this platform would provide the most expedient approach to understanding the homogeneity or heterogeneity of response across oncogene and targeted-therapy pairs. We investigated treatments that had shown clear evidence of clinical benefit or at least promising preliminary efficacy in the proposed eligibility genotype in any tumor histology. We describe the frequency with which actionable genetic alterations occurred in a US population of 5,954 patients with advanced refractory cancer.

## PATIENTS AND METHODS

The study was designed and co-administered by the Division of Cancer Treatment and Diagnosis, NCI, and the ECOG-ACRIN Cancer Research Group (ECOG-ACRIN) with the participation of the NCI National Clinical Trials Network and NCI Community Oncology Research Program (Data Supplement).

### Patient Selection

Adult patients (age ≥ 18 years) with any solid tumor, lymphoma, or myeloma that had progressed on standard treatment or without prior therapy if no curative treatment existed were eligible for screening. There was no limitation on the number of prior treatments (Data Supplement). The NCI-MATCH study (ClinicalTrials.gov identifier: NCT02465060) was approved by the NCI Central Institutional Review Board, the institutional review board of record for all participating institutions. All patients signed a written informed consent document.

### Selection of Drugs

Treatments included in NCI-MATCH were single agents or combinations, FDA-approved or investigational, and required to have a recommended phase II dose as well as a molecular alteration that might predict response on the basis of preclinical or clinical data, and at least provisional evidence of clinical activity (Data Supplement).

### Definition of Molecular Alterations

Actionable molecular alterations in the NCI-MATCH clinical trial met one of the definitions outlined in the Data Supplement and the rates of actionable alterations reported here reflect all biomarker and therapy pairs regardless of our ability to gain access to the investigational agents (the only relevant case being *IDH* mutations). The next-generation sequencing (NGS) platform also surveyed an expanded set of tumor-suppressor genes and other oncogenes either associated with therapeutic resistance or for which we did not have a targeted agent included in the NCI-MATCH subprotocols (eg, *IDH1/2* mutations).

### Biopsy Specimens and Tumor Profiling

Patients were required to have either a clinically indicated or low-risk core needle biopsy specimen (risk of severe adverse event, < 2%). Tumor that was removed for clinical indication within the prior 6 months, without a response to subsequent therapy, was permitted. All specimens were shipped for processing to the central Clinical Laboratory Improvement Amendments–accredited Tissue Qualification Laboratory at MD Anderson Cancer Center (MDACC). Medical Dictionary for Regulatory Activities coding provided by the accruing institution was used for pathologic classification. The tumors of all patients who were accrued to a treatment arm had central review of pathology classification performed at the ECOG-ACRIN Central Biorepository and Pathology Facility at MDACC and were coded with International Classification of Diseases for Oncology 3.1.

### NGS and Immunohistochemistry Assays

Central NGS assay was an adapted Oncomine AmpliSeq panel (ThermoFisher Scientific, Waltham, MA) performed as reported previously.^12^ The assay was performed on the Ion Torrent PGM or S5 and sequenced 143 genes and > 4,000 annotated variants, including single nucleotide variants (SNVs), insertion and deletions, amplifications, and selected translocations, with a minimum read depth of 500×. The assay achieved concordant results in all four competitively chosen NCI-MATCH Network Clinical Laboratories: (1) the MDACC Molecular Diagnostics Laboratory; (2) Massachusetts General Hospital Center for Integrated Diagnostics; (3) Yale Clinical Molecular Pathology Laboratory; and (4) the Molecular Characterization Laboratory at the Frederick National Laboratory for Cancer Research.^12^

The MDACC Clinical Immunohistochemistry Laboratory performed immunohistochemistry for PTEN expression, for nuclear expression of MLH1 and MSH2, and for Rb expression if cases matched to a cyclin inhibitor.^13^ All assays also received approval from the New York State Department of Health, for which the MDACC was the permitted laboratory.

### Comparison of NCI-MATCH Molecular Alterations to The Cancer Genome Atlas

To assess descriptively whether SNV frequency and type were similar in the relapsed/refractory advanced cancer population of the NCI-MATCH trial and primary tumors, we compared NCI-MATCH sequencing results with those of The Cancer Genome Atlas (TCGA)^14^ for seven tumors that were assessed in both databases (Data Supplement).

## RESULTS

### Demographics

NCI-MATCH opened on August 12, 2015; 1,117 sites registered 6,391 patients until registration for centralized molecular screening closed on May 22, 2017 (Data Supplement). Patient characteristics are summarized in Table 1. The four most common tumors in the US population (NSCLC, breast, colorectal, and prostate cancers) represented 37.5% of the accrual (Table 2; Data Supplement). The median number of prior therapies was three, and < 25% of patients had no or one prior therapy.

**TABLE 1.**
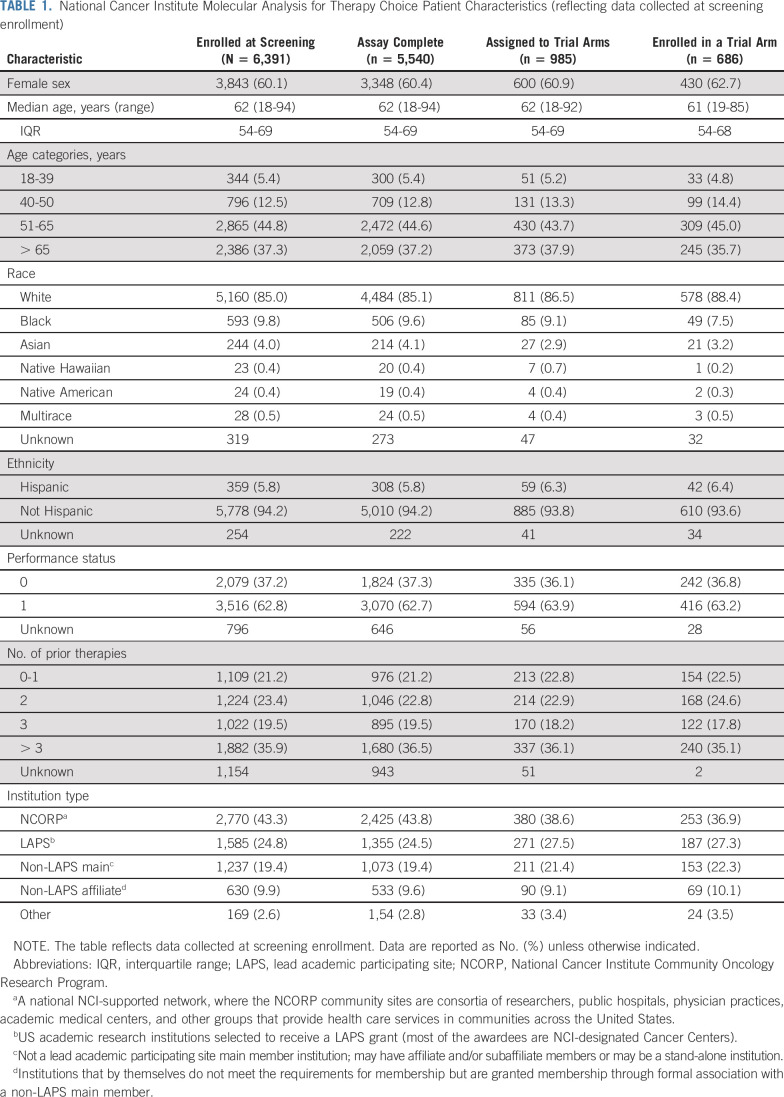
National Cancer Institute Molecular Analysis for Therapy Choice Patient Characteristics (reflecting data collected at screening enrollment)

**TABLE 2.**
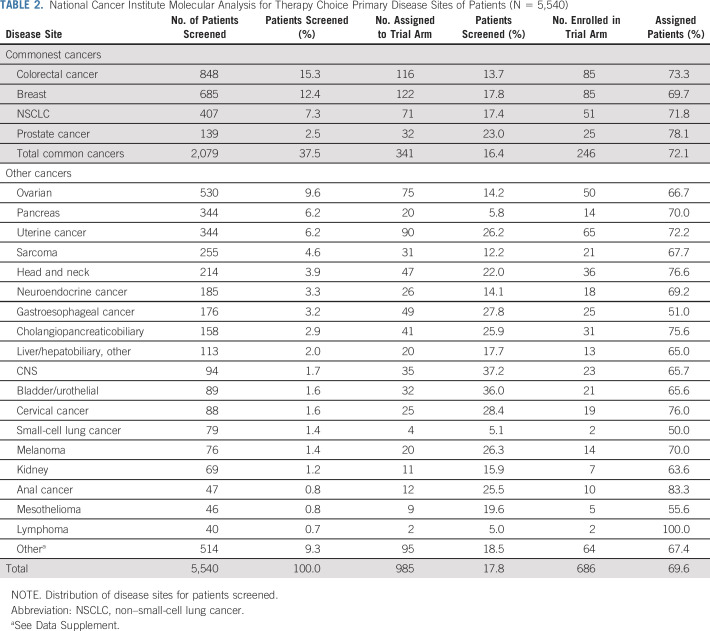
National Cancer Institute Molecular Analysis for Therapy Choice Primary Disease Sites of Patients (N = 5,540)

### Biopsy and Assay Performance

Of 6,391 registered patients, 5,954 submitted a tumor specimen. Of these, 4,629 patients had a biopsy performed specifically for NCI-MATCH screening, 1,211 had an archived specimen from prior tumor biopsy or excision, and source of tissue was unknown for 121 patients. Adverse consequences of the screening biopsy were assessed within 30 days after the biopsy (Data Supplement). Of 4,627 patients with data, 26 (0.6%) and seven (0.2%) experienced grade 3 and 4 events, respectively. No deaths were related to the biopsy procedure. Of the 5,954 samples submitted, 5,540 (93.0%) were sequenced successfully. Molecular testing could not be performed on some tumors for a variety of different reasons. Absence of or insufficient amount of tumor in the specimen and/or presence of only necrotic tumor explained the vast majority. The median and 75th percentile times from receipt of samples to return of results were 16 days and 23 days, respectively.

### Treatment Subprotocol Assignment Rates

The subprotocols available at some time during the screening portion of the trial are summarized in the Data Supplement. Molecular alterations for assignment to an NCI-MATCH subprotocol were present in 37.6% of patients (Data Supplement). When molecular, prior treatment, and specific cancer exclusions were accounted for, the match rate was 26.4%. Lack of subprotocol availability, because the subprotocol had reached accrual or because of the limit on certain histologic types was reached, led to a treatment assignment rate of 17.8% (n = 985 of 5,540; 95% CI, 16.8 to 18.8).

Assignment rates for NSCLC, colorectal, breast, and prostate cancer were 17.4%, 13.7%, 17.8%, and 23.0%, respectively (Table 2). Assignment rates > 25% were found in patients with CNS cancer (37.2%), urothelial cancer (36.0%), cholangiocarcinoma pancreaticobiliary (25.9%), cervical cancer (28.4%), gastroesophageal cancer (27.8%), melanoma (26.3%), uterine cancer (26.2%), and anal cancer (25.5%). Conversely, assignment rates were low in patients with pancreatic cancer (5.8%), small-cell lung cancer (5.1%), and lymphoma (5.0%; Table 2).

Seventy percent of assigned patients received treatment on a subprotocol. Eleven of 30 subprotocols reached the accrual goal of at least 31 eligible patients. No subprotocol whose targeted alteration had a prevalence of < 1.5% reached the accrual goal in this phase of the trial (18 subprotocols).

### Distribution of Genomic Alterations and Co-Occurring Potential Resistance Alterations

**FIG 1.**
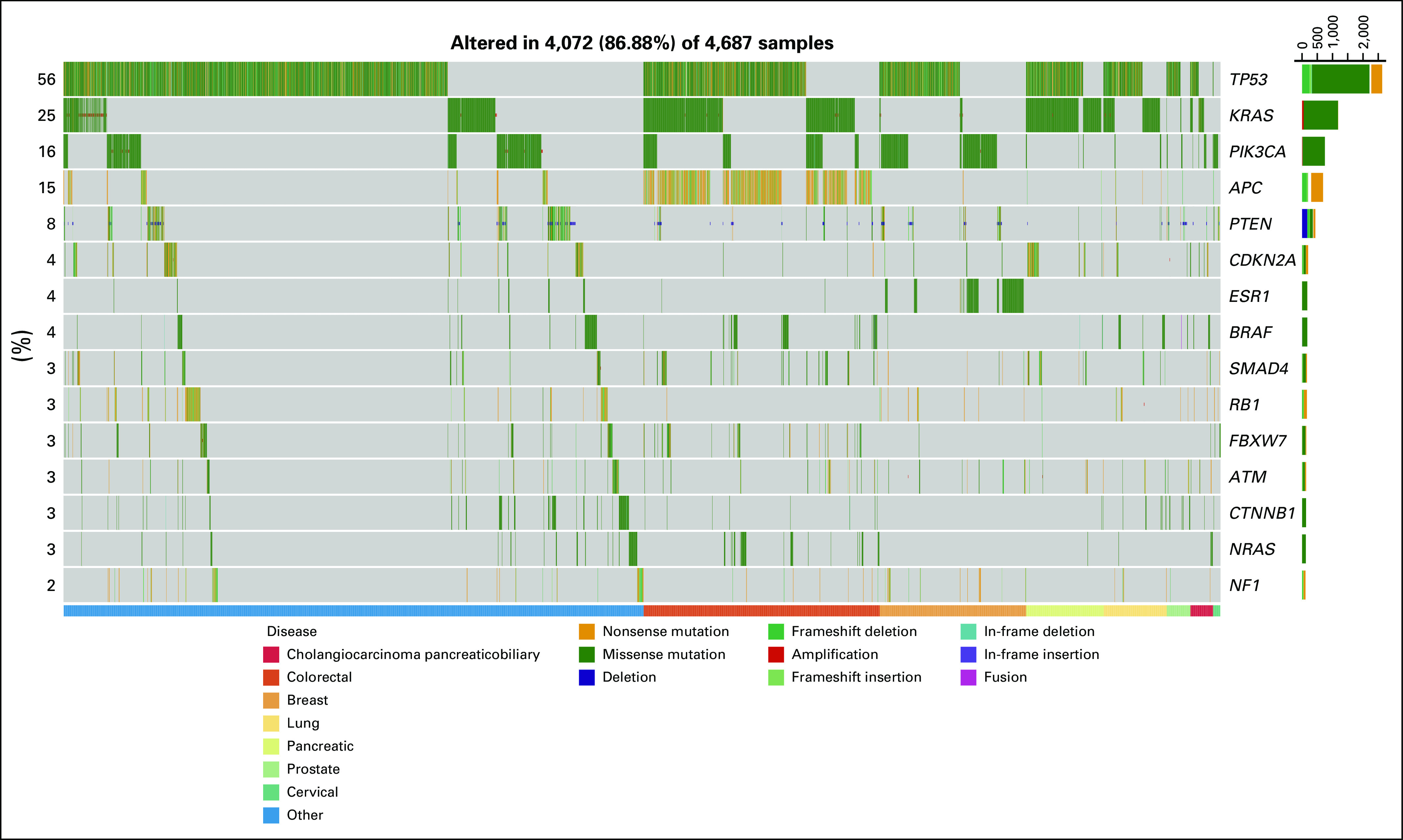
Gene alterations observed in National Cancer Institute Molecular Analysis for Therapy Choice trial. A genomic waterfall plot displaying the top 15 most frequently mutated genes ranked by their rate of mutation and categorized by variant type—amplification, deletion, missense, nonsense, in-frame insertion, in-frame deletion, frameshift insertion, frameshift deletion, and fusion. The lower bar categorizes the alterations based on seven disease types and combines all others. For the purposes of this figure, negative *PTEN* expression by immunohistochemistry is assigned the deletion variant type.^28,29^

Deleterious or activating mutations in *TP53* (47.4%), *KRAS* (21.2%), and *APC* (12.4%)^15,16^ were commonly observed. The most prevalent co-occurring mutations were *KRAS* and *TP53* in 12.1% of tumors. The most frequently observed actionable alterations were in *PIK3CA* (11.8%) and *PTEN* (6.3%); all other actionable alterations were observed in ≤ 3% (Fig 1, showing only those patients with a mutation of interest). Patients with single eligible alterations and without other actionable or nonactionable alterations (34.1%) were hypothesized to be the most likely to be responsive to the assigned targeted therapy.

Of patients with the most commonly identified actionable alterations, 37.6% (n = 723 of 2,083) were excluded from treatment due to co-occurring mutations known to confer resistance. For example, patients whose tumors had *PIK3CA* alterations were excluded from an NCI-MATCH treatment addressing these mutations if there were co-occurring *RAS* or *PTEN* resistance-conferring alterations (31.3% of *PIK3CA* mutant cases). However, 42.2% of these patients had co-occurring alterations in *TP53* that did not preclude treatment assignment but could potentially cause resistance.

Of patients assigned to NCI-MATCH subprotocols, 54.0% of patients with actionable alterations also had co-occurring mutations in tumor-suppressor genes that have been implicated in therapeutic resistance in settings such as PI3Kα inhibitors in *PIK3CA*-mutant cancers and BRAF inhibitors in *BRAF*-mutant melanoma and colorectal cancer (*TP53*, 45.0%; *KRAS*, 13.8%; or *APC*, 12.5%), but these did not preclude treatment assignment.^17,18^ Activating mutations in genes that also contribute to oncogenesis—*ERBB2, CCND1*, and *NF1*—were found in 13.2% of patients. In patients assigned to the NCI-MATCH subprotocol addressing *ERBB2* mutations, 29.3% had a co-occurring *TP53, PIK3CA*, *KRAS, EGFR*, or *PTEN* loss or mutation that did not preclude assignment but potentially could contribute to resistance.

### Comparison of Molecular Alterations in NCI-MATCH and TCGA

**FIG 2.**
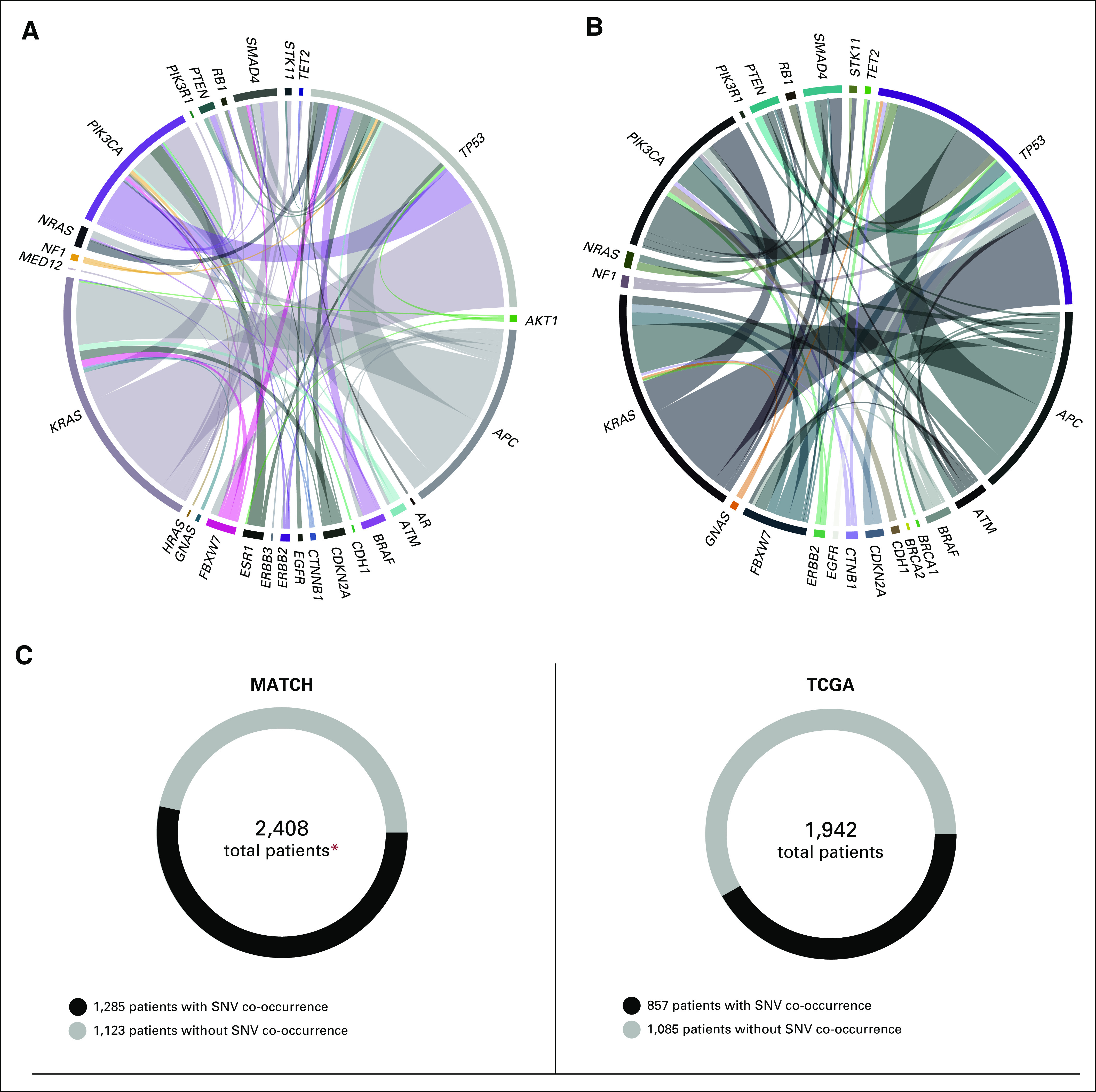
Co-occurring single nucleotide variants (SNVs) in the National Cancer Institute Molecular Analysis for Therapy Choice (NCI-MATCH) trial and The Cancer Genome Atlas (TCGA). (A, B) Circle plots depict the co-occurrence of mutations across seven tumor types compared (cervical squamous carcinoma, colorectal adenocarcinoma, cholangiocarcinoma-pancreaticobiliary, invasive breast carcinoma, lung adenocarcinoma, prostate adenocarcinoma, and pancreatic carcinoma). Band thickness represents the fraction of co-occurrence within each of NCI-MATCH and TCGA. For the purposes of these figures, only mutations in gene pairs occurring in more than five patients have been plotted. *BRCA* gene co-occurrences in NCI-MATCH were present just below the applied threshold. (C) Circular figures represent the total number of patients versus patients with or without SNV co-occurrence. (*) Patient must have one of the top seven diseases.^28,29^

We hypothesized that the genetic complexity of the NCI-MATCH cohort, with a median of three lines of prior, mostly cytotoxic, chemotherapy, would be far greater than that seen in the largely untreated primary tumors of TCGA. However, we found that the frequencies of the 10 genes most commonly harboring mutations (SNVs) in the seven tumor types compared across both cohorts were broadly similar (Data Supplement). We found that 52.8% of NCI-MATCH tumors had co-occurring mutations, as contrasted with 44.1% for TCGA (Fig 2). The frequency of *TP53* mutations and *RAS* mutations was numerically greater in the NCI-MATCH cohort for each histology, as has been observed in other populations with advanced cancer.^19,20^ However, these alterations did not correlate with number of previous treatments (Data Supplement).

**FIG 3.**
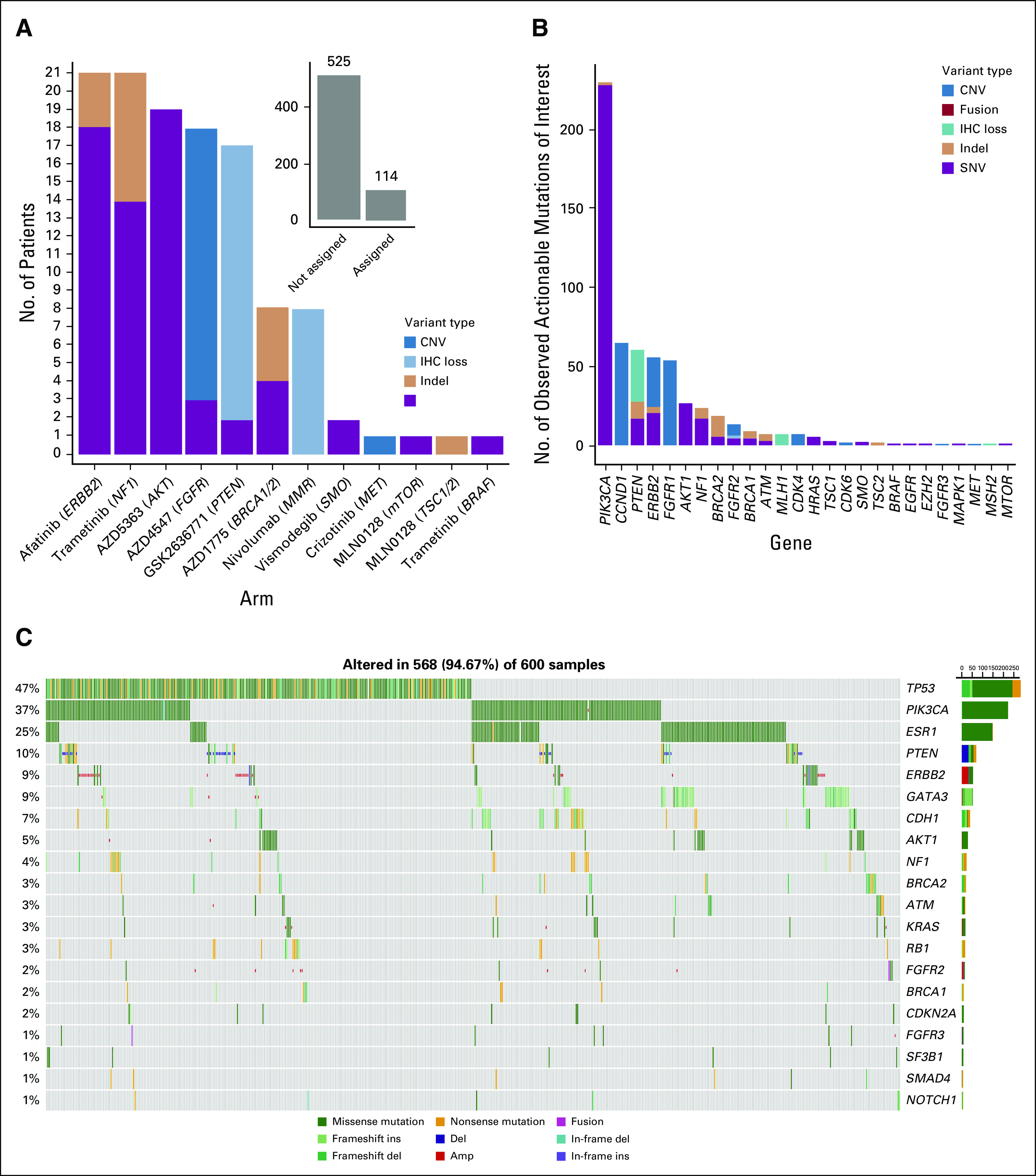

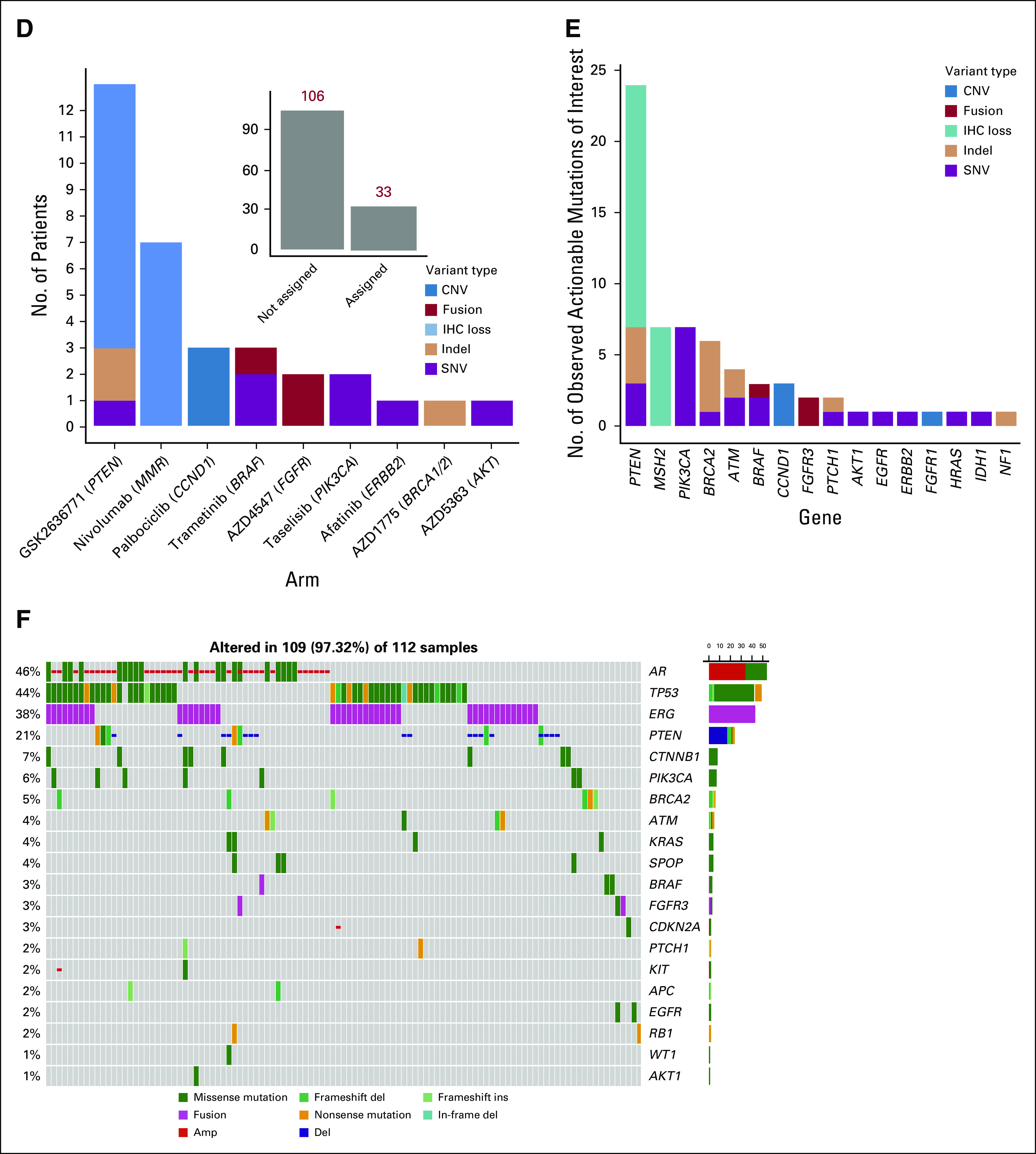
(A-F) Treatment assignments, actionable alterations, and gene alteration by disease cohort. (A) Number of patient assignments by agent/target and variant type for invasive breast cancer (inset displays patients assigned *v* not assigned in this cohort). (B) Number of observed actionable alterations by gene and variant type in patients with breast cancer; includes targetable gene mutations lacking a National Cancer Institute Molecular Analysis for Therapy Choice agent. (C) A genomic waterfall plot displaying the top 20 most frequently mutated genes ranked by their rate of mutation and categorized by variant type (amplification [amp], deletion [del], missense, nonsense, in-frame insertion [ins], in-frame deletion, frameshift insertion, frameshift deletion, and fusion) in patients with breast cancer. For the purposes of this figure, negative PTEN expression by immunohistochemistry (IHC) is assigned the deletion variant type. (continued on following page)

We then looked for evidence of genetic evolution specifically in tumor types for which molecularly targeted therapies are broadly applied. Indeed, we detected frequent androgen receptor alterations (46%) in prostate cancer and estrogen-receptor alterations (25%) in breast cancer (Fig 3). *EGFR* T790M mutations were found in nearly half of the patients with *EGFR*-mutant NSCLC who had previously received EGFR-inhibitor therapy.

We noted the greatest difference between the frequency of actionable alterations in cholangiocarcinoma cases in TCGA and NCI-MATCH, likely a consequence of our much larger sample size in NCI-MATCH (n = 153) versus that of TCGA (n = 23). NCI-MATCH data confirm that these patients had tumors rich in molecular targets^21^ for which investigational agents are available, as follows: *IDH1* mutations (17%*), CDKN2A* mutations (10%), *BRAF* mutations (7%), *ERBB2* alterations (6%), *NRAS* mutations (6%), *IDH2* mutations (5%), and *FGFR2* alterations (3%), which segregated largely by site of origin of the cholangiocarcinoma. Intrahepatic cholangiocarcinoma had an assignment rate of 29.1% to 12 different NCI-MATCH subprotocols.

## DISCUSSION

To our knowledge, NCI-MATCH represents the first attempt to establish the likelihood of identifying targeted investigational therapeutic options within a clinical trial cohort representative of the population of patients with advanced refractory cancer. We demonstrated the feasibility of nationwide accrual of patients with successful biopsy at local facilities, a high rate of technical success using a specific NGS platform addressing most of the relevant molecular alterations found in cancers, timely return of results, and, if eligible, protocol treatment. A stringent definition of actionable molecular alterations for which promising investigational agents existed was developed and 37.8% of patients had an actionable alteration in their tumor, similar to an analysis of a large sample of patients from individual major academic medical centers.^20,22^ When exclusion criteria were applied to this broad population with advanced cancer, for whom ≤ 30 treatment subprotocols were available, the assignment rate was 17.8%.

Specificity of the NGS assay was prioritized, and minimum variant allele and copy number values were put into place to optimize that feature. The assay reported on a finite gene list that was fit for purpose to identify targets for the agents selected for the trial and was deemed more than adequate to support required inclusionary actionable variants as well as exclusionary variants required for the trial. Resulting limitations include the limited number of and breadth of coverage of genes, which reduced the potential to characterize off-target pathways of effects or resistance. Fusions were detected by sequencing RNA of known and previously reported fusions; the assay was not capable of detection of novel fusion variants. The lower rate of severe complications from the required screening tumor biopsy procedures (< 1% grade 3 or 4 events; no fatal complications) was achieved by providing guidelines directed to interventional radiologists in the protocol and a scoring system to qualify metastatic lesion by location, size, and radiographic features as predicted safe sites for core needle biopsy (Data Supplement).

The match rate varied widely across tumor types. More than 25% of patients with cholangiocarcinoma, melanoma, and cancers of the prostate, uterus, gastroesophageal junction, urothelium, CNS, or cervix matched to a treatment. By contrast, only 5.8% of those with pancreatic cancer did, reflecting the lack of targeted treatments for the common mutations in this group of patients. Among the tumor profiles we compared with those in TCGA, cholangiocarcinoma stands out as a tumor type with the most actionable alterations. Given that standard treatments for this tumor are few, exploration of targeted treatments in multiarm master protocols may aid development of more effective treatment of this group of patients with poor prognosis.

NCI-MATCH differs from other recently reported NGS-guided clinical trials in several respects. Some studies have focused on exploring targeted agents approved in at least one tumor type across other cancer types when routine clinical NGS testing identifies such patients.^23,24^ Single-center trials have generally used molecular tumor boards to adjudicate the off-label use of approved agents or triaging to available clinical trials, with widely varying rates of treatment assignment.^25,26^ The largest reported multicenter trial sought to evaluate the benefit of offering therapies based on characterization of hormone receptor expression and genetic alteration in the PI3 kinase and MAP kinase pathways compared with physicians’ choice of therapy. Forty percent of patients were assigned to one of 10 available treatment regimens on the experimental arm (n = 293 of 741 patients).^27^ To our knowledge, NCI-MATCH is the only trial that used uniform, centralized molecular testing and a rules algorithm to assign treatments systematically based on predetermined molecular eligibility, rather than a tumor board. Unlike other studies evaluating the clinical utility of NGS, NCI-MATCH was designed to investigate both approved and investigational agents broadly across cancer types, beyond established biomarker-defined indications.

Most of the actionable alterations in NCI-MATCH occurred at frequencies of < 5%, consistent with other analyses.^19,20^ The striking responses occasionally observed with drugs targeting rare mutations such as *NTRK* fusions motivate broad screening across cancer types, but the logistics of conducting research in these populations in a single trial are daunting. The investigation of novel therapies in less common molecular subgroups is made more efficient by investigating many therapies in parallel in an NGS-guided platform trial. For alteration frequencies < 1.5%, however, a much larger screening population is needed to identify enough patients to enroll a typical, single-arm, phase II trial cohort. For these molecular subgroups, we have now used external academic and commercial sequencing platforms that are being used in routine practice, to continue accrual in NCI-MATCH, and will confirm the outside NGS result with the NCI-MATCH assays.

The requirement for fresh tumor biopsy specimens was driven by the desire to have an accurate assessment of tumor somatic genetic make-up at the time of study entry. This is not necessary for the purpose of detecting founder or truncal mutations that are present in all tumor cells but was considered important for the purpose of identifying additional genetic changes that manifest during tumor evolution and are enriched under selective pressure of other therapies.

Among those tumors with an actionable alteration and available investigational agent in NCI-MATCH, co-occurring alterations in genes with evidence that they mediate resistance in other settings (ie, *TP53*, *KRAS*, or *APC*) or that activate potentially competing survival pathways were common. At the time NCI-MATCH was designed, these co-alterations lacked evidence of degradation of response to the specific targeted therapies and did not preclude treatment assignment to many of the subprotocols. For patients with *TP53* and *APC* mutations (62% harbored at least one of these alterations), indirect therapeutic strategies are needed to address the consequences of loss of function in these critical tumor-suppressor mechanisms.^15,16^ Although we have not yet compared the NCI-MATCH specimens with the patients’ primary tumors, the available data from TCGA and NCI-MATCH suggest there is not substantial evolution in the genetic features from primary to metastatic cancers. Furthermore, surprisingly, cytotoxic chemotherapy does not significantly alter the genomic landscape. Pending the results of ongoing whole-exome sequencing of patients who were assigned to treatment subprotocols, we cannot yet conclude whether important information is gained from tumor biopsy specimens at baseline versus sequencing of archival tumor specimens. Therapies that place significant selective pressure (eg, hormonal therapy for prostate and breast cancer; EGFR inhibitors in *EGFR*-mutated NSCLC) do produce clear, pathway-convergent resistance mutations.

Our findings support the feasibility and efficiency of using NGS to triage patients to investigational therapy, provided that a sufficiently large pool of agents is made available. The molecular landscape of a population of patients with relapsed, refractory advanced cancer strongly endorses a shift to investigation of combination targeted-therapy regimens in such genetically complex tumors, most notably those harboring multiple actionable alterations.
